# Mesoporous Silicon with Modified Surface for Plant Viruses and Their Protein Particle Sensing

**DOI:** 10.3390/s8106225

**Published:** 2008-10-01

**Authors:** Yuriy Vashpanov, Jung Young Son, Kae Dal Kwack

**Affiliations:** 1 Research Institute of Electrical and Computer Engineering, HIT Hanyang University, 17 Haengdang-dong, Seongdong-ku, 133-791, Seoul, Korea; 2 School of Computer and Communication Engineering, Daegu University, 15 Naeri, Jinryang, Geyeongsan, Gyeongbuk 712714, Daegu, Korea

**Keywords:** Biosensors, Porous Silicon, Plant Viruses, MOSFET, Double Gate, current-voltage response

## Abstract

Changes in electric parameters of a mesoporous silicon treated by a plasma chemical etching with fluorine and hydrogen ions, under the adsorption of NEPO (Nematodetransmitted Polyhedral) plant viruses such as TORSV (Tomato Ringspot Virus), GFLV (Grapevine Fan Leaf Virus) and protein macromolecule from TORSV particles are described. The current response to the applied voltage is measured for each virus particle to investigate the material parameters which are sensitive to the adsorbed particles. The peculiar behaviors of the response are modeled by the current-voltage relationship in a MOSFET. This model explains the behavior well and the double gate model of the MOSFET informs that the mesoporous silicon is a highly sensitive means of detecting the viruses in the size range less than 50 nm.

## 1. Introduction

Biosensors based on nanotechnologies are now widely investigated for the possible applications to monitor environmental pollutants [1]. Detecting plant viruses in agriculture is one of such applications. Since various shapes, sizes and biochemical properties of a large number of different viruses and bacteria are already known [2], the biosensors need to be able to identify different types of biological objects. In this direction, the porous silicon is now widely considered as a candidate for the biosensors[3].

It is known that viruses are having sizes from 20 to 500 nanometers and bacteria having more than 500 nanometers. Among the viruses, plant viruses, in particular NEPO plant viruses such as GFLV and TORSOV have the orbicular shape with a diameter about 30 nanometers. Each viral particle, i.e., a virion consists of a genetic core and a protective protein coat called viral protein, i.e., capsid [2]. The capsid does not have a smooth surface. Its surface has many protuberances. These protuberances are composed of polar organic molecules. It is the polar molecules which make the viral protein adhere very well to various material surfaces. Therefore viral particles' interaction with the surfaces is granted by property of the viral proteins' macromolecule structure.

Mesoporous silicon (MesoPS) is a good material for sensing biological objects because it can sense bio-substance, and is bio-compatible, mechanically stable and simple to use [4-8]. Furthermore, it doesn't need analyte molecules as in carbon nanotube [9]. Porous structure has a similar to nanoporous membrane for the filtration of virions with an ultrahigh selectivity [10]. Therefore pores in porous silicon work as a natural membrane for small biological particles. It turned that, the pore structure was found to play a significant role during infiltration of protein into the photonic crystal [11].

It was found that the porous silicon gone through a plasma-chemical process involving fluorine and hydrogen ions can induce more polar organic molecules to be absorbed and have more stable electric parameters [12]. Hence the treatment increases the sensitivity of the porous silicon to the adsorbed viruses.

In this paper changes in electric parameters of porous silicon under the adsorption of plant NEPO-viruses particles are described. Protein macromolecules from TORSV viruses are used to determine the role of the coat protein in the adsorption process.

## 2. The preparation of a MesoPS silicon sensor and loading of a virus onto the sensor

Samples of MesoPS were formed from a p-Si wafer in 100 direction by the anode electrochemical etching within HF-based solution [HF(48%):ethanol=1:1]. During etching, additional illumination is provided and ultrasonic processing is applied to the silicon surface. After manufacturing, a plasma chemical treatment with ions of fluorine and hydrogen has been done on the surface of the MesoPS. The average porosity of the MesoPS and the diameters of the registered pores has been controlled to be about 47 % of the upper surface area and not to exceed 100 nanometers. Detailed processes of preparing the MesoPS are described in the reference [12]. Measurements with a secondary ionic mass spectroscope have shown that the concentrations of hydrogen, oxygen and fluorine ions in the MesoPS are in the ranges of 15%, 1% and 4% of total atomic mass, respectively. Ions of fluorine and hydrogen stabilize the electrical properties of the MesoPS: The doped high-energy hydrogen ions on the surface area of the MesoPS results in increasing the saturated number of unbalanced silicone molecules. Since fluorine is more active to some oxygen molecules, it deters the surface structure changes of the MesoPS when it is open to air.

**Figure 1.**
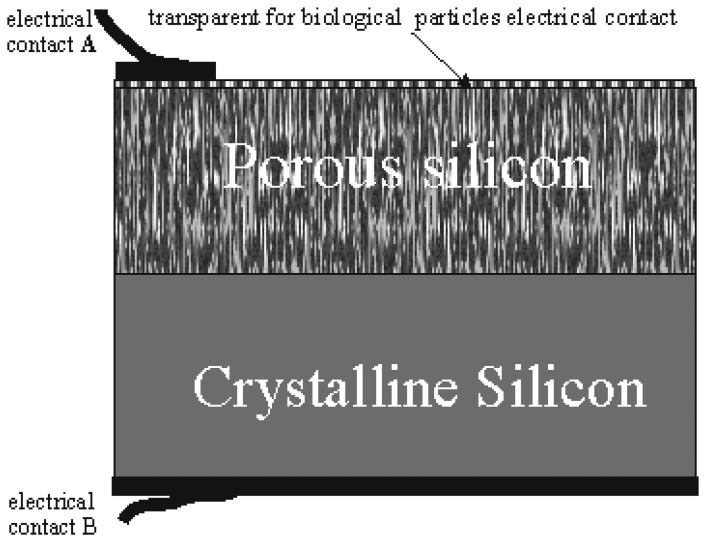
Cross-sectional view of the microelectronic devices with electric contacts A and B on the base of the mesoporous silicon formed from crystal silicon.

Figure 1 shows the profile of the MesoPS sensors for the plant viral particle detection. On a part of the top surface of the porous silicon a thin metal layer is deposited as the electrode. The electric contact A is transparent for biological particles. The external voltage is applied through the bottom of the substrate and the electrode. The sensors can be considered as composed of many silicon wires with lengths more 500 nm and thicknesses in nano-dimensions, covered with a thin dielectric layer containing fluorine and hydrogen atoms. Charge transport will be probable only through each nanowire between contacts A and B (figure 1). Current-voltage characteristics of the sensors between electric contacts A and B are measured before and after loading the viral particles.

TORSV (Tomato Ringspot Virus) and GFLV (Grapevine Fan Leaf Virus) viruses and protein particles are prepared by the department of microbiology and virology of I.I.Mechnikov university in Odessa, Ukraine. The protein particles are prepared by removing the core in the center of each TORSV virus.

**Figure 2.**
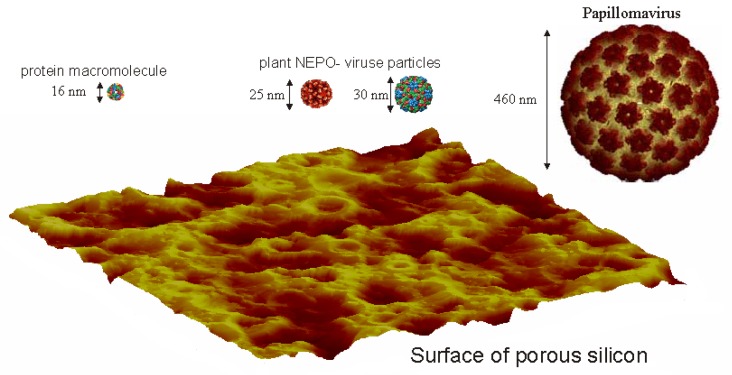
Atomic force microscopic image of mesoporous silicon surface and images of protein macromolecule, NEPO plant virus and papilloma virus particles. The numbers represent their characteristic sizes.

Loading the viral particles on the sensors has been carried out by the standard procedure [13]: Each viral particle is put to a bowl with a twice-distilled water to be the final concentration of 1 mg/ml. This solution is placed in dialysis devices and dialysised against the twice-distilled water with 100 times of the solution in volume, for a day at temperature 8 to 10 °C. Then a porous silicon sensor is placed in the solution for one hour under 10 mm Hg vacuum at 25°C. Finally the sensor is dried in a desiccator with CaCl2 for two hours. In figure 2, an atomic force microscopic image of mesoporous silicon surface and images of protein macromolecule, plant NEPO-viruses and papilloma virus particles. It is obvious that due to geometrical parameters of the viruses, they will be selectively getting into the pores. I.e., NEPO-viruses and their protein macromolecule can get into the pores as shown in Figure 2.

**Figure 3.**
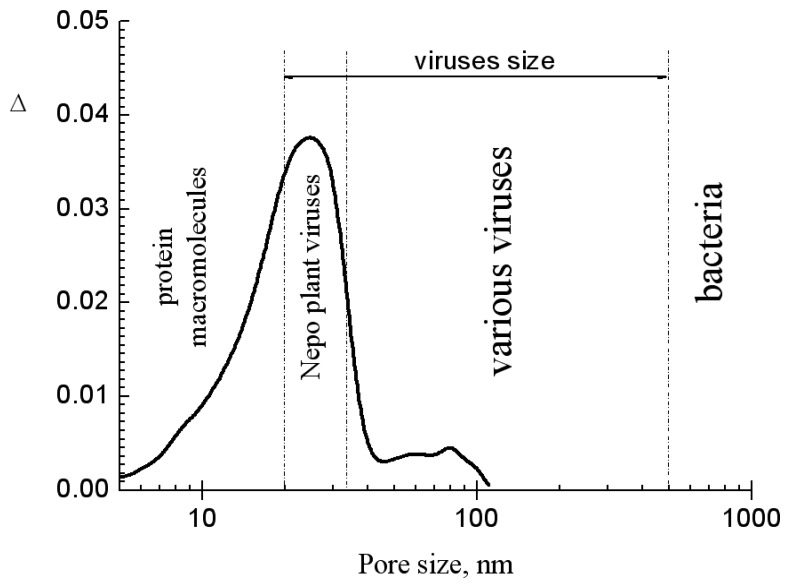
Pore size distribution of the MesoPS sensors and characteristic sizes of viruses, bacteria, and protein macromolecules.

In figure 3, the pore size distribution of the MesoPS sensors and the characteristic sizes of viruses, bacteria, and protein macromolecules are shown. The sensors' pore sizes are mostly less than 50nm in diameter. Hence the sizes are too small to accept virus particles with sizes bigger than 100 nm and bacteria. Thus plant NEPO-virus particles can be selectively adsorbed by the pores. The geometrical sizes of the plant virus particles and the protein macromolecule are good for the pore sizes. It means that the MesoPS sensor can selectively detect different NEPO-viruses based on their geometrical sizes. Hence on geometrical size point of view, the MesoPS sensor is very effective in detecting viruses with sizes not more than 50 nanometers.

## 3. Results and Discussion

**Figure 4.**
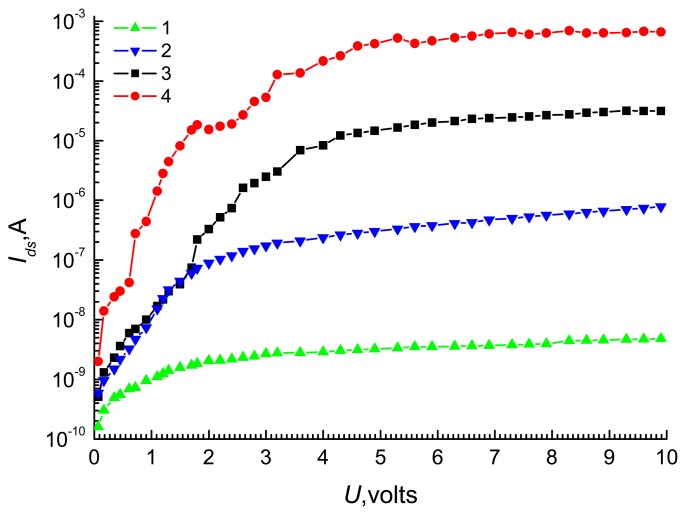
Dependences of current *I_ds_* on bias *U* before (curve 1) and after adsorbing TORSV and GFLV viruses, and protein particles (curves 2 to 4).

Figure 4 shows the dependences of current *I_ds_* on the bias *U* with before (curve 1) and after adsorbing TORSV and GFLV viruses, and protein particles for curves 2-4, respectively. The all 3 sensors show the same characteristics when no viruses are adsorbed as shown in curve 1. The dependence of current *I_ds_* on bias *U* for all curves in Fig. 3 is very similar to source-drain characteristics of the MOSFET (Metal-oxide semiconductor field emission transistor). The curves reveal that filling pores with different size particles work like different voltages applied between source and gate in the MOSFET. The adsorbed biological particles have almost the same chemical properties, but difference in their geometrical sizes. It is obvious that the number of the viruses/proteins in each particle will be inversely proportional to its geometrical size. Hence particles with larger sizes will introduce more charges to the sensor. Hence they introduce more influences to the electrical parameters of the sensor.

The non-monotonic increase in current with U could be explained by assuming that each silicon wire in the MesoPS is a MOSFET with a different physical size. Each MOSFET reveals different electrical characteristics from others. The total current density in quantum wires and other quantum devices are represented by the sum of currents originated from quantum and drift diffusion phenomena. In these wires and devices, the Current-Voltage characteristics are also showing non-monotonic relationships [14]. The physical sizes are equally significant to both classical and quantum physics. Dimensional quantization leads to the occurrence of discontinuities in *I* – *U* curve. The current transport theory in quantum wires explains the non-monotonic current-voltage relationship in figure 3 [14].

**Figure 5.**
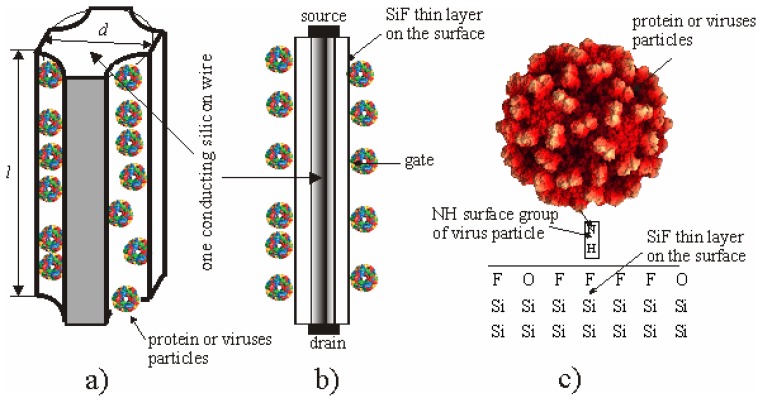
Illustrations of filling of a porous silicon wire surface with the adsorbed virus particles (a) and (b), and interaction between polar molecules on the surface of a capsid and the F-Si-H group on porous silicon surface (c).

The top electrode plays the role of a source and the crystalline silicon plays the role of a drain (figure 5). Since the silicon wires are covered by the dielectric SiF layer, the surface with adsorbed particles can plays the role of a gate (figure 5b). The viruses adhere easily to the SiF layer because ions such as fluorine and oxygen on the surface of the SiF layer interact with polar molecules at the surface of the viral proteins.

The current *I_ds_* can be given as sum of current *I_dsi_* through each MOSFET which is symbolizing each silicon wire between electrodes.

(1) $$I_{ds}=\sum_{i=1}^{N}I_{\text{dsi}}\approx NI_{\text{dsi}}$$

where *N* represents the total number of the wires between electrodes. In Eq. 1, it is assumed that the current in each wire is equal to those of other wires. The number *N* in the geometrical size of 1 mm^2^ if it is assumed that the average distance between pores are ≈100 nm, becomes equal to 108. If it is assumed that all these wires have almost the same characteristics, the total current will be 108 times of the current through each wire, i.e., 10^8^ *I_dsi_*.

Geometrical and physical parameters of a nanodimensional transistor are similar to the DG (Double Gate) device described by Taur [15].

In the DG device, the current *I_dsi_* can be derived from the following relationships:

(2) $$I_{\text{dsi}}=I_{0}[\beta \tan\beta -0.5\beta^{2}+r\beta^{2}\tan^{2}\beta ],\;\text{and}\;I_{0}=\mu \frac{W}{L}\frac{4{ɛ}_{Si}}{t_{Si}}{\left(\frac{4kT}{q}\right)}^{2}$$

In Eq. 2, *β* is obtained as the solution of the following equations.

(3) $$\ln\beta -\ln(\cos\beta )+2r\beta \tan\beta =v\;\text{and}\;v=\frac{q(V_{g}-\Delta \phi -V)}{2kT}-\ln(\frac{2}{t_{Si}}\sqrt{\frac{2{ɛ}_{Si}kT}{q^{2}n_{i}}})$$

where *q* is a electron charge, *k* is Boltzmann constant, *T* is absolute temperature, *ε_Si_* is dielectric constant of silicon, *t_Si_*, *L* are channel thickness and length respectively (fig.5, b), *W* is the device width, *μ* the effective mobility, Δϕ is the work function difference at the gates, *V_g_* and *V* are the gate voltage and the applied source drain voltage, *n_i_* is intrinsic carrier concentration in silicon and *r* = *ε_Si_*δ/*ε_ox_t_Si_*, where *ε_ox_* is dielectric constant of the SiF layer, is structural parameter. The dependence β and *I_ds_* on *V_g_* is found at H. Moris's work [16]. Function ln *I_ds_*(*V_g_*) is linear from 0 up 0.4 volts.

**Figure 6.**
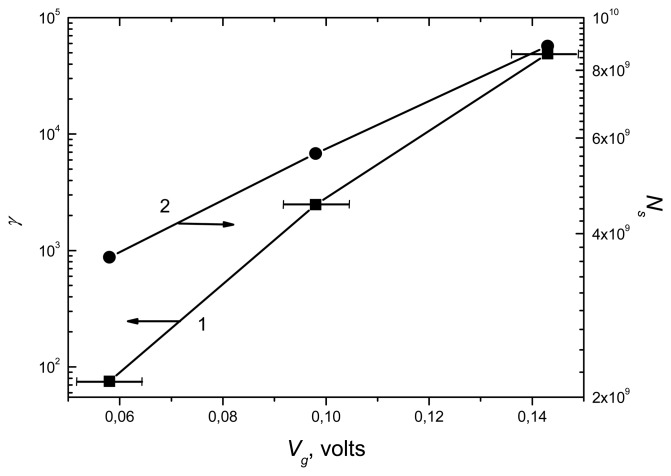
Dependences of parameter *γ* (curve 1) and adsorbed number of particles, *N_s_* (curve 2) on the gate voltage *V_g_*. Error bars represent confidence intervals.

To find equivalent *V_g_* values for curve 2 to 4 in Fig. 4, let's introduce *γ* which is defined as,

(4) $$\gamma =\frac{I_{ds}^{ad}}{I_{ds}^{0}},$$

where $I_{ds}^{0}$ and $I_{ds}^{ad}$ are saturation currents before (curve 1 in Fig. 4) and after adsorbing biological particles (curve 2-4 in Fig. 3). For curve 1, it is possible to consider that *V_g_* is approximately equal to zero. By this consideration, *V_g_* value for after adsorbing TORSV and GFLV viruses, and TORSV protein particles can be calculated by the *γ* values corresponding to each of them. The magnitude of *γ* is in the range from 102 up to 105 after adsorbing TORSV and GFLV viruses and protein particles (Fig.4 curves 2 to 4). This informs that detecting biological nano-objects with the MesoPS is a highly sensitive method. In Fig. 6, curve 1 shows *V_g_* values calculated from parameter *γ*, which obtained from Fig. 4 data for the applied source drain voltage of 5 volt.

For a purpose, it might be necessary to count the number of the adsorbed particles. This can be done by estimating the capacitance of the sensor based on its geometry and material characteristics. Since each silicon wire has the form of a coaxial cylinder by the dielectric layer etched on its surface as shown in Fig. 2(b), can be used to estimate the capacitance formula [17]:

(5) $$C_{i}=\frac{2\pi ɛ{ɛ}_{0}L}{\ln\frac{r_{2}}{r_{1}}}=\frac{2\pi ɛ{ɛ}_{0}L}{\ln\frac{r_{1}+\delta }{r_{1}}}$$

where *ε*_0_ is the permittivity of vacuum. The capacitance C*_i_* for inner radius *r*_1_ =25 nm, surface dielectric layer thickness *δ*=5 nm, length L=0.5 μm and the dielectric constant ε=4.5 [18] is equal to 10-7 nF. Hence the capacitance of the sensor with active surface area of 1 mm^2^ is calculated as around ≈10 nF from Eq. 5.

When it is assumed that the charge on each electrically active particle is equal to one electron charge, the number of adsorbed particles can be estimated as following way: Since the surface charge *Q_s_* can be expressed as *Q_s_* = *V_g_C*, the number of adsorbed particles, *N_s_* are calculated as *N_s_* = *Q_s_*/*q* = *V_g_C*/*q*. In Fig. 6, curve 2 shows the dependence of number *N_s_* on voltage *V_g_* from experimental data.

The volume of the total porous space of the MesoPS with porosity 47% is calculated as 4.7·10^−13^m^3^. Since the volumes of protein, GFLV and TORSV virus particles are calculated as 4.096·10^−24^ m^3^, 1.63·10^−23^m^3^, and 2.7·10^−23^m^3^, respectively, with the diameter values shown in Fig. 1. The maximum numbers of the protein, the GFLV and the TORSV particles, which can fill the volume are estimated as 1.47·10^11^, 3.0·10^10^ and 1.74·10^10^, respectively. These numbers are more than the *N_s_* in 10^9^ range as specified in curve 2 of Fig. 6. This means that the porous space is a partially filled. These numbers also indicate that the smaller size particles can penetrate more to the porous space. As a consequence, the conduction response of MesoPS increases, i.e., more current is induced for smaller size particles as shown in Fig. 4. Hence it can be said that the MesoPS has a very high sensitivity to the viral and their protein particles. MesoPS is performing a role of natural membranes. This role will probably be responsible for raising selectivity of the MesoPS. It is expected that the selectivity of MesoPS for protein particles will be increased if the pore sizes are less than 20 nm.

It is also noticed that as informed by Eq. 5 and the MOSFET model, the capacitance value of the sensor is defined only by its geometrical dimension and material property. The presence of viral and protein particles in the pores cannot result any change in the capacitive parameters of the sensor because no changes in chemical compositions and geometrical properties of the sensor are induced by the particles.

## Conclusions

Electronic devices based on the mesoporous silicon can be used to detect selectively small biological particles such as TORSV and GFLV which are classified as the plant NEPO viruses. The mesoporous silicon sensors can effectively select viruses with sizes mostly less than 50 nanometers among huge number of known viruses. The manufacturing simplicity is the advantage of the mesoporous silicon sensor. Improving manufacturing technology, it is possible to achieve selective identification of various biological particles and their concentration.
